# Corrugator Muscle Activity Associated with Pressure Pain in Adults with Neck/Shoulder Pain

**DOI:** 10.3390/medicina60020223

**Published:** 2024-01-28

**Authors:** Takahiro Yamada, Hiroyoshi Yajima, Miho Takayama, Konomi Imanishi, Nobuari Takakura

**Affiliations:** Department of Acupuncture and Moxibustion, Tokyo Ariake University of Medical and Health Sciences, Tokyo 135-0063, Japan; takahiro.yamada@tau.ac.jp (T.Y.); yajima@tau.ac.jp (H.Y.); takayama@tau.ac.jp (M.T.); imanishi.konomi@tau.ac.jp (K.I.)

**Keywords:** pain, neck/shoulder pain, pain assessment, pressure pain, corrugator muscle, facial expression, visual analogue scale, algometer

## Abstract

*Background and Objectives:* No studies have reported corrugator muscle activity associated with pain in people with pain. This study aimed to develop an objective pain assessment method using corrugator muscle activity with pressure pain stimulation to the skeletal muscle. *Methods:* Participants were 20 adults (a mean ± SD age of 22.0 ± 3.1 years) with chronic neck/shoulder pain. Surface electromyography (sEMG) of corrugator muscle activity at rest (baseline) and without and with pressure pain stimulation applied to the most painful tender point in the shoulder was recorded. Participants evaluated the intensity of the neck/shoulder pain and the sensory and affective components of pain with pressure stimulation using a visual analogue scale (VAS). The percentages of integrated sEMG (% corrugator activity) without and with pressure pain stimulation to the baseline integrated sEMG were compared, and the relationships between the % corrugator activity and the sensory and affective components of pain VAS scores were evaluated. *Results:* Without pressure stimulation, an increase in corrugator muscle activity due to chronic neck/shoulder pain was not observed. The % corrugator activity with pressure pain stimulation was significantly higher than that without stimulation (*p* < 0.01). A significant positive correlation between corrugator muscle activity and the affective components of pain VAS scores with pressure stimulation was found (ρ = 0.465, *p* = 0.039) and a tendency of positive correlation was found for the sensory component of pain VAS scores (ρ = 0.423, *p* = 0.063). *Conclusions:* The increase in corrugator muscle activity with pressure pain stimulation to the tender point in adults with chronic neck/shoulder pain was observed, although increased corrugator muscle activity resulting from the chronic neck/shoulder pain was not. These findings suggest that corrugator muscle activity with pressure pain stimulation can be a useful objective indication for tender point sensitivity assessment in the skeletal muscle with pain.

## 1. Introduction

Pain is associated with various diseases. To control pain, an objective assessment of pain in conjunction with a subjective pain assessment may be of great help in providing appropriate treatment [1]. The International Association for the Study of Pain (IASP) defined pain as “An unpleasant sensory and emotional experience associated with, or resembling that associated with, actual or potential tissue damage” [2]. According to this definition, pain is primarily a subjective sensation based on one’s own experiences. Therefore, it is difficult for healthcare providers to objectively assess patients’ subjective pain. Under such circumstances, the current pain assessments used in many clinical settings and research fields are subjective self-completion methods, such as the visual analogue scale (VAS), numerical rating scale (NRS), face rating scale (FRS), and verbal rating scale (VRS). Among these subjective pain assessments, VAS is the most frequently used in clinical settings and pain research because the results obtained using VAS are considered to be the most accurate and reproducible [3,4].

However, patients are required to understand the meaning of VAS and have the ability to assess their pain using VAS by imagining the most severe pain, which is very vague and difficult. Furthermore, patients’ standards for pain assessment vary, making it difficult for health providers to accurately evaluate individual patients’ pain and compare pain intensities between patients [5,6,7]. It has been pointed out that, even when accurate assessments can be made, medical providers tend to underestimate patients’ pain in subjective assessments such as VAS in the clinical field, which may lead to the neglect of necessary care [8]. Therefore, the development of objective measures to compensate for subjective pain assessments is required.

Currently, the objective assessment of pain from patients’ facial expressions has been gaining attention in the development of new pain assessment methods because facial expressions expressing unpleasantness are often observed as an indication of pain intensity in patients in clinical fields [9]. The specific facial muscles that express pain are the corrugator, orbicularis oculi, levator labii superioris, zygomaticus major, and risorius muscles [10,11], which were identified using experimental pain. Furthermore, a study using surface electromyography (sEMG) to examine the relationship between pain and facial muscles concluded that the corrugator muscle is the most relevant to the expression of pain among the facial muscles, as the activity of the corrugator muscle increases as the intensity of the pain increases [12,13,14]. Based on these reports, we studied corrugator muscle activity associated with pressure pain stimulation applied to the tender point in the shoulder in healthy adults with no pain. In this previous study, we reported that corrugator muscle activity significantly increased with pressure pain stimulation, and its activity was correlated with sensory and affective components of pain VAS scores [15]. To date, the reported results on the relationship between subjective assessment methods such as VAS and the sEMG activity of facial expressions have only been obtained from healthy adults [12,13,14,15].

In the clinical field, pressure stimulation is often used in palpation to examine musculoskeletal pain [16], which is experienced by many people around the world [17]. Among musculoskeletal disorders, neck/shoulder pain is one of the most common complaints worldwide [18]. Nonspecific neck/shoulder pain, not from specific diseases, was the second most common chief complaint in a comprehensive survey conducted in Japan in 2022 [19]. Pain from musculoskeletal disorders causes economic losses via a decrease in performance at work, including absenteeism due to treatment for pain and other activities [20,21,22,23,24]. Furthermore, musculoskeletal pain has a strong impact on daily life, as well as work, and indirect economic losses have been recognized [25]. The treatment of musculoskeletal pain is extremely important not only for achieving physical and psychological benefits and improving the quality of life of patients but also for reducing economic losses to society. To achieve appropriate pain treatment, it is necessary to evaluate a patient’s pain from multiple perspectives, as pain consists of sensory, cognitive, and affective-motivational dimensions [26,27,28,29,30]. Therefore, an accurate and informative assessment of the effects of pain treatments may be achieved by measuring both the sensory and affective components of pain.

Therefore, we investigated corrugator muscle activities associated with pressure pain stimulation in chronic musculoskeletal pain-aware adults in the neck and shoulder to develop an objective index for pain assessment.

## 2. Methods

This study was approved by the Ethics Committee of Tokyo Ariake University of Medical and Health Sciences (Tokyo Ariake University of Medical and Health Sciences, approval no. 366, date of approval: 17 December 2021). The research methodology was based on that of a previous study conducted on healthy participants who had no pain [15].

### 2.1. Participants

A total of 20 adults (10 males and 10 females; with an age of 22.0 ± 3.1 years; height of 163.9 ± 9.0 cm; weight of 59.3 ± 12.5 kg (mean ± SD)) with nonspecific chronic neck/shoulder pain due to muscle stiffness participated in this study. The duration of neck/shoulder pain was 795.1 ± 691.1 (mean ± SD) days. Adults with pain due to musculoskeletal disorders including cervical spondylosis, cervical hernia of the intervertebral disk, cervicobrachial disorder, thoracic outlet syndrome, cerebrovascular disease, and neurological disorders were excluded. Before the study started, we explained the details of this research to the participants orally and provided a written explanation, stating that they could withdraw at any point after consenting to participate in the research of their own free will without any consequences. They provided informed consent to participate in this study.

### 2.2. Pressure Stimulation

#### 2.2.1. Point to Apply Pressure Pain Stimulation

Pressure pain stimulation was applied at the point where the pain was the most strongly elicited when pressure stimulation was applied with the fingertip across the trapezius, levator scapulae, and rhomboid muscles [31] (hereafter called “the tender point”), which are known to be associated with neck and shoulder pain. The tender point was chosen because we often palpate the most painful point with pressure stimulation to examine and treat musculoskeletal pain in the clinical field [16].

#### 2.2.2. Pressure Pain Threshold

An algometer (Algometer Type II, SBMEDIC Electronics, Stockholm, Sweden) was used to measure the pressure pain threshold, as in a previous study [15]. This algometer displays the pressure intensity digitally, which makes it possible to apply pressure to the body at a constant rate of increase in pressure per second [32].

Pressure was applied to the tender point with a 0.5 cm^2^ circular probe at a 50 kPa increase per second starting from a pressure of 10 kPa, which elicited no pain. After the pressure stimulation started, the participants pushed a button connected to the algometer to stop the measurement when they felt pain to determine their pain thresholds. The pressure pain threshold was measured three times for each participant, and the mean value was used to determine the individual pain threshold.

#### 2.2.3. Pressure Pain Stimulation

The intensity of the pressure pain stimulation was twice that of the pressure pain threshold in each participant. A researcher well trained in the usage of the algometer manually applied pressure pain stimulation to the tender point in the shoulder for 5 s using the algometer.

### 2.3. Measurement of Surface Electromyography (sEMG) of the Corrugator Muscles

**Figure 1:**
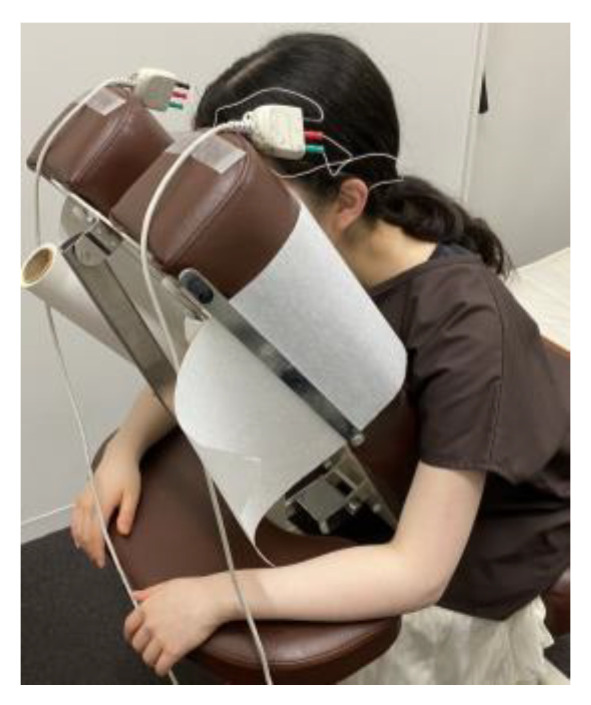
Experimental setting. Participants on a massage chair with electrodes attached to the forehead to record corrugator muscle activity.

Each participant sat facing down on bilateral pads used for head support on a massage chair (Takada Bed Massage Chair, Quick Massage Round Chair N Type/TB-519, Osaka, Japan) without the center of the face around the corrugator muscle being touched during the sEMG recording (Figure 1).

To record the activity of the corrugator muscle, surface electrodes (NSC electrode, NM-317Y3, Nihon Kohden Corporation, Tokyo, Japan) were attached to a crossing point between a vertical line extending upward from the medial corner of the eyelid and a horizontal line passing through the upper edge of the eyebrow, and to another point 10 mm lateral from this crossing point on both sides of the face. Ground electrodes were attached to the left and right mastoid processes.

The sEMG of corrugator muscle activity was recorded using an electromyogram (Neuropack X1: MEB-2306, Nihon Kohden Corporation, Tokyo, Japan), with a sampling frequency of 1000 Hz and a bandpass filter of 20–500 Hz, utilizing the bipolar derivation method according to previous studies [14,15,33,34].

Analysis software (Labchart, ADInstruments, Nagoya, Japan) was used to process the recorded sEMG. The recorded sEMG of corrugator muscle activity was A/D-converted every ms, fully rectified, and integrated. The integrated sEMGs of every measurement on both sides of the corrugator muscle were averaged for statistical analysis. Each mean of the integrated sEMGs on both sides without and with pressure pain stimulation was then divided by the mean of the baseline integrated sEMGs at rest, and the resulting values are expressed as percentages (hereafter called “% corrugator activity”).

### 2.4. Subjective Sensory and Affective Component of Pain

First, the intensity of chronic neck/shoulder pain with no stimulation applied was assessed using a 100 mm VAS (0: no pain; 100: maximum imaginable pain). The same scaled VAS was used to measure the intensity of the sensory component of pain during pressure pain stimulation. In addition, the intensity of the affective component of pain (unpleasantness) was measured using a 100 mm VAS (0: not unpleasant; 100: maximum imaginable unpleasantness). Further, a 100 mm VAS (0: not pleasant; 100: maximum imaginable pleasantness) [15,35] was used to assess the intensity of pleasantness if a participant felt pleasantness when they received pressure pain stimulation.

### 2.5. Experimental Procedures

The participants sat on a massage chair in a relaxed position. The skin of the forehead of the participants was sterilized with 70% alcohol-soaked cotton prior to the surface electrodes being attached to the above-mentioned points on the face.

A researcher located and marked the tender point and measured the pressure pain threshold using the algometer. First, when the sEMG of the corrugator muscle activity became stable, the sEMG of the baseline corrugator muscle activity at rest was recorded for 5 s. Then, the sEMG was recorded without pressure stimulation for 5 s. Subsequently, another researcher applied pressure pain stimulation at twice the intensity of the pressure pain threshold to the tender point in the participants using the algometer for 5 s. During the pressure pain stimulation, sEMGs of corrugator muscle activity were recorded. After completing the pressure stimulation, the participants assessed the intensity of the sensory and affective components of pain, including pleasantness, on VAS.

### 2.6. Data Analysis

Statistical analyses were performed using SPSS Statistics Version 29 (IBM Japan, Ltd., Tokyo, Japan). The Wilcoxon signed-rank test was used for between-group comparisons of % corrugator activity without vs. with pressure pain stimulation and of the intensity of neck/shoulder pain vs. sensory component of pain with pressure stimulation on VASs. For the pressure pain threshold and baseline % corrugator activity, the Mann–Whitney U test was used for between-group comparisons in the participants with chronic neck/shoulder pain in this study vs. in healthy participants without pain in our previous study [15]. Spearman’s correlation coefficient was used to analyze the relationship of % corrugator activity, sensory and affective components of pain intensities during pressure pain stimulation, and neck/shoulder pain intensity on VASs. As some participants felt pleasantness when they received pressure pain stimulation, the pleasantness VAS scores were expressed as negative values; then, we translated the intensities of the unpleasantness or pleasantness of all participants as an affective component of pain VAS scores, which range from −100, maximum imaginable pleasantness, to 0, neither pleasant nor unpleasant, and 100, maximum imaginable unpleasantness.

## 3. Results

### 3.1. Neck/shoulder Pain Intensity, Pressure Pain Threshold, and Baseline Corrugator Muscle Activity

The intensity of the chronic neck/shoulder pain in the twenty participants was 47.7 (mean) ± 15.1 (45.0) (SD (median)) on VAS.

For the pressure pain threshold in the participants, the mean ± SD (median) was 292.1 ± 137.2 (272.5) kPa, which was significantly less than 531.3 ± 192.5 (493.0) kPa in the healthy adults without chronic neck/shoulder pain in the previous study [15]. There was no significant correlation between the pressure pain threshold and the intensity of the chronic neck/shoulder pain.

The mean ± SD (median) of baseline corrugator muscle activity at rest in the participants with neck/shoulder pain in this study was 15.0 ± 6.8 μV (13.0), which was not significantly different from that of 13.4 ± 2.4 μV (13.3) in the healthy adults without pain in the previous study [15].

### 3.2. Corrugator Muscle Activity without and with Pressure Pain Stimulation

**Figure 2:**
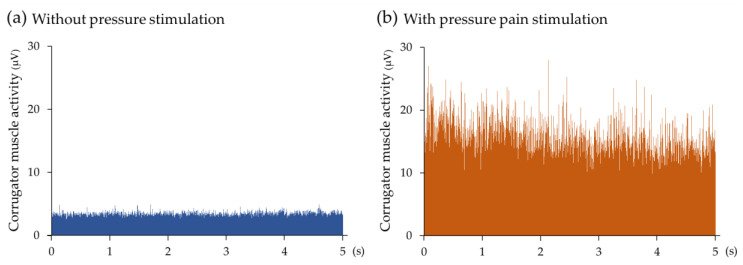
The ensemble average of rectified surface electromyography (sEMG) of corrugator muscle activity (**a**) without (blue) and (**b**) with pressure pain stimulation (orange) to the tender point for 5 s in 20 chronic neck/shoulder pain participants. The means of rectified sEMGs of both sides of the corrugator muscles in each participant, which were generated via rectified sEMGs converted from bilateral sEMGs, were used to determine the ensemble average in order to depict the average activity curve of the corrugator muscle. The vertical and horizontal axes represent muscle activity and time, respectively.

Figure 2 shows the ensemble average of a sEMGs of full-wave rectified corrugator muscle activity without and with pressure pain stimulation applied to the tender point of the shoulder in all participants. With the application of pressure stimulation, large corrugator muscle activity was observed, although little corrugator muscle activity was observed without pressure stimulation in the participants with neck/shoulder pain.

**Figure 3:**
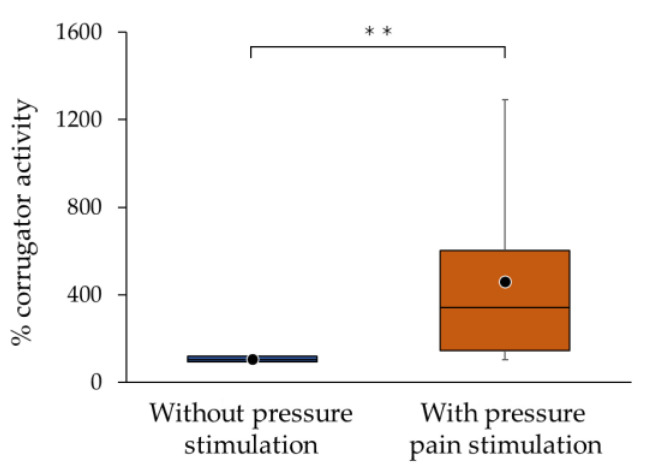
Corrugator muscle activity in 20 chronic neck/shoulder pain participants without (blue) and with pressure pain stimulation (orange). The “% corrugator activity” indicates the percentage of the integrated surface electromyography (sEMG) without and with pressure pain stimulation to the baseline integrated sEMG. The bottom, middle, and top lines in the boxes correspond to the 25th, median, and 75th percentiles, respectively. The whiskers extend from the 10th to 90th percentiles. The circles indicate arithmetic means. There was a significant difference between without and with pressure pain stimulation. ** *p* < 0.01.

In the sEMG recordings of all participants, the % corrugator activity with pressure pain stimulation (mean ± SD (median), 461.0 ± 411.4 (342.4)) was significantly larger than that without pressure stimulation (105.5 ± 15.8 (103.8)) (*p* < 0.01) (Figure 3). Furthermore, the % corrugator activity with pressure pain stimulation was 4.32 ± 3.75 (3.34) times that without pressure pain stimulation in 20 participants. In 2 out of 20 participants, the value was less than 1.1 times.

### 3.3. Sensory and Affective Component of Pain Intensities with Pressure Pain Stimulation

**Figure 4:**
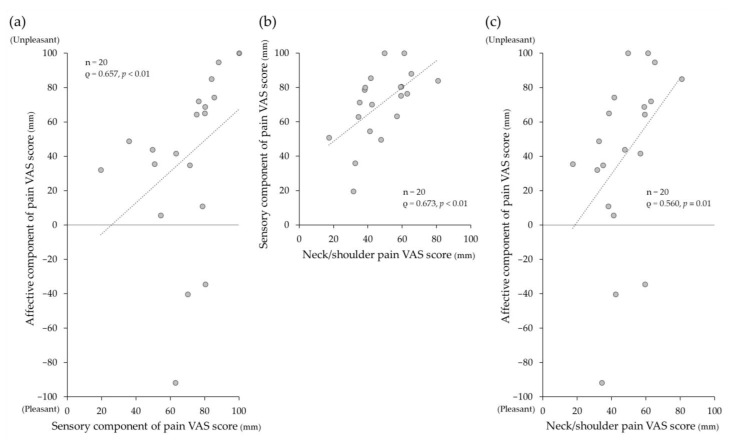
(**a**) Correlations between the intensity of sensory and affective components of pain with pressure pain stimulation, and correlations between the intensity of (**b**) sensory and (**c**) affective components of pain with pressure pain stimulation and chronic neck/shoulder pain on visual analogue scale (VAS). There were significant positive correlations between them. The uppermost dot represents two data points in (**a**).

The mean ± SD (median) of the sensory component of pain with pressure pain stimulation was 70.4 ± 20.3 (75.9), which was significantly larger than the chronic neck/shoulder pain of 47.7 ± 15.1 (45.0) (*p* < 0.01) on VAS. The mean ± SD (median) of the affective component of pain VAS scores with pressure pain stimulation was 40.6 ± 50.3 (46.3). Three participants reported pressure pain as “comfortable” (pleasantness VAS scores: 63.0, 80.5, and 70.1). There was a positive significant correlation of the intensity between sensory and affective components of pain (ρ = 0.657, *p* < 0.01) (Figure 4a).

Significant positive correlations were found between the intensity of chronic neck/shoulder pain and the sensory component of pain (ρ = 0.673, *p* < 0.01) (Figure 4b) and between the intensity of chronic neck/shoulder pain and the affective component of pain (ρ = 0.560, *p* < 0.01) (Figure 4c) on VAS.

In two participants, the % corrugator activity with pressure pain stimulation was 1.03 and 1.01 times that without pressure stimulation, which means that corrugator muscle activity during pressure pain stimulation did not increase; however, their sensory component of pain VAS scores were 63.3 and 19.6, and their affective component of pain VAS scores were 41.6 and 32.1, respectively.

### 3.4. Relationship between Corrugator Muscle Activity and Pain Intensities

**Figure 5:**
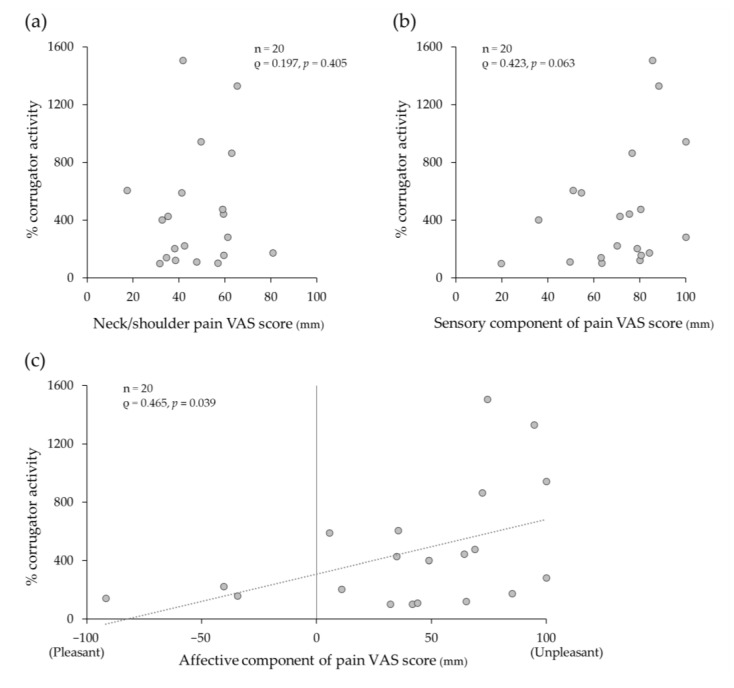
Correlation between corrugator muscle activity and the intensity of (**a**) chronic neck/shoulder pain, (**b**) sensory component of pain with pressure pain stimulation, and (**c**) affective component of pain with pressure pain stimulation on visual analogue scale (VAS). “% corrugator activity” indicates the percentage of the integrated surface electromyography (sEMG) without and with pressure pain stimulation to the baseline integrated sEMG. A significant positive correlation was found between corrugator muscle activity and the intensity of the affective component of pain.

No significant correlation was observed between % corrugator activity with pressure pain stimulation and the chronic neck/shoulder pain VAS score (ρ = 0.197, *p* = 0.405) (Figure 5a). There was no significant correlation between % corrugator activity and the sensory component of pain VAS score with pressure pain stimulation (ρ = 0.423, *p* = 0.063) (Figure 5b), but a significant positive correlation was found between % corrugator activity and the affective component of pain VAS score with pressure pain stimulation (ρ = 0.465, *p* = 0.039) (Figure 5c).

### 3.5. Adverse Events

No adverse events were observed.

## 4. Discussion

As the establishment of an objective pain assessment is required, the recording of sEMGs of corrugator muscle activity with pain was investigated as a pain assessment method in this study. In this context, we investigated sEMGs of corrugator muscle activity with pressure pain stimulation in healthy adults and found that it significantly increased with pressure pain stimulation. In the present study, we assessed corrugator muscle activity and sensory and affective components of pain intensities using VAS with pressure pain stimulation to the tender point in participants who were aware of musculoskeletal pain in the neck and shoulder. Corrugator muscle activity with pressure pain stimulation was significantly higher than that without pressure stimulation, and there was a significant positive correlation between corrugator muscle activity and the affective component of pain VAS scores; that is, larger corrugator muscle activity occurred with more unpleasantness. The results of this study suggest that corrugator muscle activity with pain induced by pressure stimulation was useful as an indicator for tender point sensitivity assessment in the skeletal muscle with pain when an increase in corrugator muscle activity was not observed in chronic musculoskeletal pain.

Without any stimulation, only little activity in the corrugator muscle was observed. Among the facial muscles said to express one’s emotions [36], the corrugator muscle is said to express pain and discomfort [10,11,12,13,14,15]. In previous studies, an increase in corrugator muscle activity was not observed when the intensity of the pain stimulus was weak and did not reach the threshold of expressing pain visible to others [37,38] to show a need for care [39], or corrugator muscle activity increased only with discomfort [33]. Based on these studies [33,37,38,39], it is assumed that the intensity of chronic neck/shoulder pain in the participants in this study was insufficient to increase corrugator muscle activity. Furthermore, the contraction of the corrugator muscles due to pain not only allows others to see the pain but also exposes one’s own weakness [40]. On the one hand, if relationships with others are not trustworthy, the corrugator muscle contraction is suppressed; on the other hand, if there is sufficient trust, it is expressed [41,42,43]. It cannot be denied that the relationship between the participants and the observer was involved in the inactivation of the corrugator muscle. Alternatively, it cannot be ruled out that the chronic musculoskeletal pain in this study had lost its fundamental role as a warning signal to induce protective responses, resulting in no corrugator muscle activity being observed.

Whatever the reasons for the corrugator muscle activity not to occur in patients with stiff neck/shoulder, there could be few cases in general clinical practice where a method for the assessment of corrugator muscle activity originating from the patient’s own musculoskeletal pain can be used. In this sense, the appearance of an increase or absence of corrugator muscle activity in patients with pain can be a criterion for determining whether the pain is severe.

Corrugator muscle activity significantly increased with pressure pain stimulation applied to the tender point in the shoulder of the chronic neck/shoulder pain participants. The intensity of the sensory component of pain with pressure pain stimulation was significantly higher than that of chronic neck/shoulder pain. These results suggest that there might be a threshold for the activation of the corrugator muscle in some neuronal circuits between the small afferent fibers conducting pain and the corrugator muscle, if any. Furthermore, the neuronal circuits between the pain afferents and corrugator muscle are assumed to be subliminally activated under a specific threshold by neck/shoulder pain, as indicated by the correlation between the intensity of chronic neck/shoulder pain and sensory component of pain on VAS with pressure stimulation, and there was a significantly lower pressure pain threshold in the chronic neck/shoulder pain participants than in healthy people without any pain in the neck or shoulder [15]. These results suggest that chronic neck/shoulder pain lowers the pain threshold, and relatively weak pressure stimulation can induce corrugator muscle activity in patients with pain but without increasing corrugator muscle activity resulting from their own pain. Considering these results, corrugator muscle activity induced by pressure pain could be a useful method to assess objective pain sensitivity of tender points in patients with musculoskeletal pain who have no increased corrugator muscle activities attributed to their own pain by measuring the threshold or magnitude of corrugator muscle activity as an indicator for assessments.

Correlations between corrugator muscle activity and either the intensity of sensory or affective component of pain with pressure stimulation were revealed in our previous study in healthy participants who had no pain [15]. In the neck/shoulder pain participants, a significant correlation was revealed only for the affective component of pain; however, there was a tendency for a positive correlation, close to a significant level, between corrugator muscle activity and sensory component of pain intensity, and there was a significant correlation between the intensity of sensory and affective components of pain. These results suggest that corrugator muscle activity expresses pain and discomfort and that it may be used for tender point sensitivity assessment in the skeletal muscle with pain. However, the affective component of pain is more likely to be involved in corrugator muscle activity than the sensory component of pain [44,45,46] considering the current results.

Although no healthy participants without neck/shoulder pain felt pleasantness with pressure pain stimulation in our previous study [15], three participants with chronic musculoskeletal pain in the neck/shoulder felt the pressure pain stimulation as pleasant in this study. This phenomenon is similar to the 28% of participants who received acupuncture, in which the skin and muscle were penetrated with a needle, and reported that the pain was pleasant [35]. This paradoxical phenomenon of perceiving pain as pleasant is thought to occur because acupuncture is recognized as a beneficial treatment [47], which may reverse the emotional sensation of pain from unpleasant to pleasant. Since the pressure pain stimulation used in this study is similar to massage stimulation, which is also recognized as a useful treatment for musculoskeletal pain, including neck and shoulder pain [48,49], it is possible that pressure pain stimulation, even with an algometer, was recognized as a beneficial treatment, similar to acupuncture. Whatever the reason, considering that healthy people without chronic musculoskeletal pain in the neck/shoulder did not feel pressure pain stimulation as pleasant [15], this phenomenon may be peculiar to musculoskeletal pain with mechanical pressure, especially chronic pain accompanied by stiffness, as we often experience it during massage treatments. This suggests the necessity of combining sensory and affective components of pain VASs with corrugator muscle activity whenever objective tender point sensitivity is assessed using pressure stimulation for musculoskeletal pain.

From the current results, there might be two ways of using corrugator muscle activity to evaluate the intensity of pain. One is for evaluating whether the pain in a patient is strong enough to activate the corrugator muscle. When corrugator muscle activity is increased in patients with pain, indicating that pain is relatively strong with unpleasantness, corrugator muscle activity can be used to evaluate pain intensity with subjective pain assessment methods. The other is for the evaluation of tender point sensitivity in the skeletal muscle with pain in patients whose corrugator muscle activity is not increased by their own pain; this is carried out by employing pressure pain stimulation to tender points to induce corrugator muscle activity. This means that corrugator muscle activity can be used to determine the effect of the treatment by applying pressure stimulation or other stimuli to induce pain before and after treatment. However, approximately 10% of the participants in our study did not show an increase in corrugator muscle activity with pressure pain stimulation, which may be a phenomenon unique to skeletal muscle pain. In such patients, sensitivity to opioids may be involved, considering that corrugator muscle activity is inhibited by opioids [50,51,52,53]. Regardless of the mechanism, corrugator muscle activity may not be indicative of pain in some people. Although the corrugator muscle cannot be used in all people, measurement of the corrugator muscle activity with pressure stimulation to tender points is expected to be used as an objective tender point sensitivity assessment in conjunction with subjective pain assessment methods to achieve a multidimensional assessment of pain; this has recently been expected because of the complexity of pain [54,55,56,57].

The pain assessment system using sEMG of corrugator muscle activity does not require the patient’s understanding of this method and thought process that is required in subjective pain assessment using VAS or NRS, to determine pain intensity by imagining the greatest pain and comparing it to the real pain [3,4,5,6,7]; thus, it may become one of the objective methods of pain assessment in clinical settings. Furthermore, considering the usefulness of this assessment system for patients who are unable to determine the amount of pain they experience [15] and the relative simplicity of utilizing this pain assessment system at the bedside [15], this system may have real clinical value.

The key findings of this study are that pressure pain stimulation to the tender point increased corrugator muscle activity in most of the participants with chronic neck/shoulder pain, whose corrugator muscle activity attributed to the original chronic neck/shoulder pain was not observed. These findings indicate that a threshold exists for activating the corrugator muscle with pain, and pressure pain stimulation activates the corrugator muscle at a lower threshold in the participants with pain. For clinical implications, in musculoskeletal pain patients whose corrugator muscle activity does not increase, corrugator muscle activity is useful as an indicator for an assessment of tender point sensitivity using pressure pain.

This study had the following limitations: All participants had pain in the shoulder but did not necessarily need treatment; they were young, healthy (except for neck/shoulder pain), and in their 20s. The results were obtained only for musculoskeletal pain. Pressure stimuli of different intensities were used across participants. The pressure pain threshold in healthy participants without pain in our previous study [15] was used for between-group comparisons of the pressure pain threshold and baseline corrugator activity in the participants with pain in this study. The possible involvement of measurement error cannot be completely ruled out with the manual algometer [58], although we used an algometer that can minimize the error by feed backing the rate of increase in pressure in real time [32], and the reproducibility of the intensities of pressures of repetitive measures by manual algometers was reported [15,59]. Further research is needed to solidify the conclusions of the present study, not only for musculoskeletal pain other than chronic neck/shoulder pain, but also for pain from other diseases and in patients with reduced facial muscle activity, such as those with facial paralysis or Parkinson’s disease.

## 5. Conclusions

Pressure stimulation applied to the tender point in the shoulder increased corrugator muscle activity in adults with chronic musculoskeletal neck/shoulder pain, although increased corrugator muscle activity resulting from chronic neck/shoulder pain was not observed. These findings suggest the usefulness of corrugator muscle activity with pressure pain stimulation as an objective indication for tender point sensitivity assessment in the skeletal muscle with pain.

## Data Availability

The data presented in this study are available on request from the corresponding author.
